# Psychological Disorders of Patients With Allergic Rhinitis in Chengdu, China: Exploratory Research

**DOI:** 10.2196/37101

**Published:** 2022-11-10

**Authors:** Heyin Huang, Yichen Wang, Lanzhi Zhang, Qinxiu Zhang, Xiaojuan Wu, Hengsheng He

**Affiliations:** 1 Department of Otolaryngology Sichuan Integrative Medicine Hospital Chengdu China; 2 Department of Geriatric Orthopedics Sichuan Province Orthopaedic Hospital Chengdu China; 3 Department of Medical Cosmetology Affiliated Hospital of Chengdu University of Traditional Chinese Medicine Chengdu China; 4 Department of Otolaryngology Affiliated Hospital of Chengdu University of Traditional Chinese Medicine Chengdu China

**Keywords:** psychological disorders, allergic rhinitis, Chengdu, China

## Abstract

**Background:**

The number of patients with allergic rhinitis (AR) has exceeded 500 million worldwide due to the unstable curative effect that can easily produce mental and psychological disorders. However, most of the relevant existing literature is one-on-one retrospective analyses or targeted meta-analyses of AR with psychological disorders like irritability, depression, and anxiety, while “multi-hospital + interdisciplinary” multiple regression analyses are scarce.

**Objective:**

This study aims to precisely identify the psychological disorders of patients with AR who were diagnosed and treated in the five most renowned hospitals in Chengdu, China over the past 5 years using 10 classification methods so as to attract attention and care from otolaryngologists.

**Methods:**

The Symptom Checklist 90 (SCL-90) was used to group and score the mental state of 827 strictly screened patients with AR according to 9 classification criteria. The scores were then compared within groups. Intergroup comparisons were made between the study group and the Chinese norm, and the positive factors for psychological disorders were extracted. Four symptoms in the study group, that is, nasal itching, sneezing, clear discharge, and nasal congestion, were scored on a visual analog scale. Partial correlation analysis was performed between the extracted positive factors for psychological disorders and the symptom scores by the multiple regression statistical method.

**Results:**

Among 827 patients, 124 (15%) had no mental health impairments, 176 (21.3%) had mild impairments, 474 (57.3%) had mild to moderate impairments, 41 (5%) had moderate to severe impairments, and 12 (1.4%) had severe impairments. The average score of the SCL-90 for all 827 patients was 2.64 (SD 0.25), which corresponded to mild to moderate mental health impairments. The 827 patients scored significantly higher for the 4 positive factors: depression, anxiety, psychosis, and other (sleep, diet). Depression was positively correlated with sneezing and clear discharge, anxiety was positively correlated with nasal itching and congestion, psychosis was positively correlated with nasal itching and sneezing, and other (sleep, diet) was positively correlated with clear discharge and nasal congestion.

**Conclusions:**

Patients with AR have mild to moderate mental health impairments, with women and those with abnormal BMI, aged ≥45 years, with a monthly salary <¥5110 (US $700), with a disease duration <13 years, residing in urban areas, with a high school or above education, or who are indoor laborers being at high risk and requiring more care, follow-up, and comprehensive therapy from otolaryngologists.

## Introduction

According to the guidelines of the World Health Organization (WHO) and Allergic Rhinitis and its Impact on Asthma, the number of patients with allergic rhinitis (AR) has exceeded 500 million globally [1]. Due to the unstable curative effect, symptoms like nasal itching, sneezing, clear discharge, and nasal congestion recur easily, which are hardly eradicated, thus readily generating mental/psychological disorders [2]. In China from 2007 to 2016, a total of 6 ear, nose, and throat doctors were hacked to death by patients with knives, and 3 doctors were seriously injured. It is thus imperative to study the correlation between AR and psychological disorders in depth, which is in line with the “biological-psychological-social” international medical model.

However, most of the relevant existing literature are one-on-one retrospective analyses or targeted meta-analyses of AR with psychological disorders like irritability, depression, and anxiety [3], while “multi-hospital + interdisciplinary” multiple regression analyses are scarce. To this end, this study makes the following 3 innovations by inviting the 5 most renowned hospitals, both Chinese and Western, in China’s Chengdu and integrating three perspectives of otolaryngology, psychology, and psychiatry. First, a comprehensive intragroup comparison is made on the study group. The 9 classification criteria for intragroup comparison are derived from the preliminary questionnaire survey, which are the factors most likely to cause psychological disorders among patients with AR that are preferentially screened by the authors. Second, an intergroup comparison is performed between the study group and the Chinese norm to derive the 4 positive factors most associated with the patients’ psychological disorders. Third, the visual analog scale (VAS) is used to score the 4 AR symptoms of nasal itching, sneezing, clear discharge, and nasal congestion, which are then subjected to the one-on-one partial correlation analysis separately with the 4 positive factors of psychological disorders.

## Methods

### Clinical Data

A total of 1536 patients with AR who received treatment at 5 hospitals from July 2013 to January 2018 were selected. A routine questionnaire survey was conducted strictly following the inclusion and exclusion criteria for the AR group, rejecting 709 cases and enrolling 827 cases.

### Ethics Approval

All selected patients signed the informed consent and questionnaire. The study has been certified by the ethics review system of the World Federation of Traditional Chinese Medicine Societies (CAP2013 (A) 0038).

### Diagnostic Criteria

We will strictly follow the latest AR diagnostic criteria promulgated by China in 2018 [4]. The following three points must be presented simultaneously: (1) having two of the four symptoms of nasal itching, sneezing, clear discharge, and nasal congestion, and symptoms last for half an hour to over an hour and occur more than 4 days weekly; (2) skin prick test parameters are positive, one of which is (++) or above, or the allergen-specific IgE is positive; and (3) the morphology of nasal mucosa shows inflammatory change.

### Exclusion Criteria

The following exclusion criteria will be used: failing to complete the questionnaire carefully or completely; having abnormal nasal anatomy (eg, those definitively diagnosed with nasal sinusitis or septum deviation); having autoimmune diseases; having systemic and chronic diseases; having any history of psychogenic illness; or having an incomplete family structure, such as separation, divorce, widowhood, and loss of child or children.

### Psychological Scale and Scoring Method

There are 90 items on the Symptom Checklist 90 (SCL-90) [5], each of which is graded from 1 to 5. The 90 items are randomly arranged and reflect 10 psychological factors.

Total average score = Total score of the 90 items / 90

Factor score = Total score of each item constituting a factor / Number of items constituting the factor

The following will be used for the classification of the symptom [6]: a factor score and total average score <1.5 points will be judged as asymptomatic, a factor score and total average score ≥1.5 points and <2.5 points will be judged as mild, a factor score and total average score ≥2.5 points and <3.5 points will be judged as mild to moderate, a factor score and total average score ≥3.5 points and <4.5 points will be judged as moderate to severe, a factor score and total average score ≥4.5 points will be judged as severe.

### Evaluation and Quantification of Symptoms

A total of 4 AR symptoms (ie, nasal itching, sneezing, clear discharge, and nasal congestion) were evaluated separately on a 10-point VAS, with 10 representing the severest, and 0 indicating the mildest.

Mean VAS score = Total scores of the five symptoms / 5

### Comparison and Research Methodologies

#### Intragroup and Intergroup Comparison

The 10 SCL-90 factor scores and whole average scores were compared between the AR group (study group) and the Chinese norm (healthy controls group) [7], from which the positive factors of the SCL-90 were extracted.

#### Partial Correlation Analysis

The 4 AR symptoms were evaluated with the VAS and recorded for scores. A partial correlation analysis [8] was made between the scores of each symptom and the positive factors through the multiple regression statistical method [9] by taking measurement data like BMI, age, monthly salary, and disease duration as the control variables.

### Statistical Analysis

If the measurement data is consistent with the normal distribution, they will be expressed by *x̅* ± *s*, and all data will accept the normality test and homogeneity test of variance in a single-sample Kolmogorov-Smirnov test. The normal distribution data was tested by independent sample *t* or paired sample *t* test, and the nonnormal distribution data was tested by Mann-Whitney *U*. A one-way and orderly contingency table will accept the rank sum test of Kruskal-Wallis *H*. Using the multivariate regression statistical method, the control variables were first established, and then a partial correlation analysis with a partial correlation coefficient was performed. SPSS 19.0 software (IBM Corp) was used to carry out the statistical analysis of the data, and the difference has statistical significance if *P*<.05.

## Results

### Intragroup Comparisons

**Figure 1:**
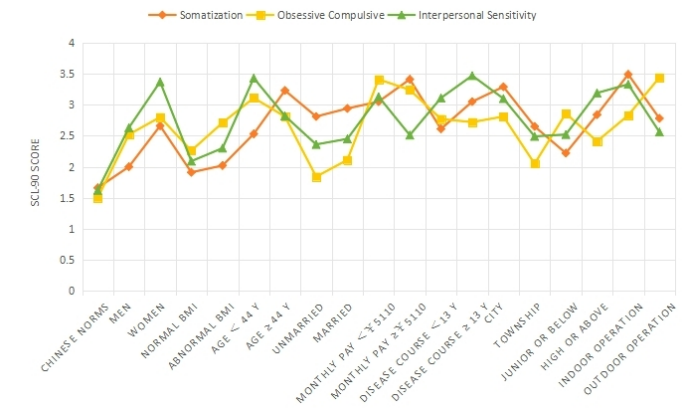
Comparison of somatization, obsessive-compulsive symptoms, and interpersonal sensitivity on 18 items in 9 groups. SCL-90: Symptom Checklist 90.

**Figure 2:**
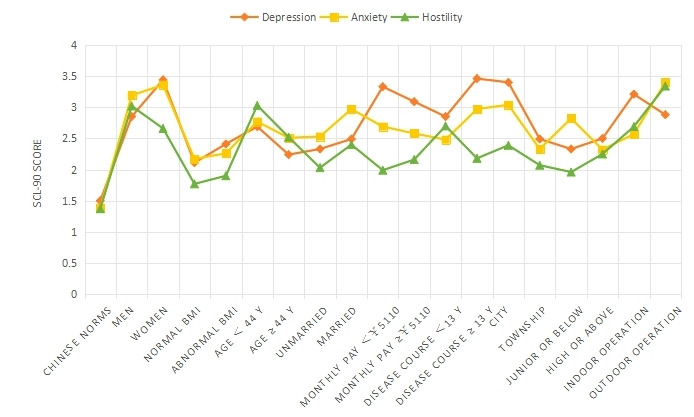
Comparison of depression, anxiety, and hostility on 18 items in 9 groups. SCL-90: Symptom Checklist 90.

**Figure 3:**
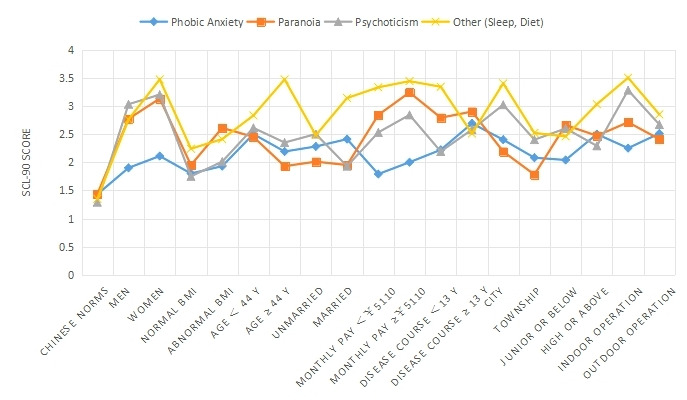
Comparison of phobic anxiety, paranoia, psychosis, and other (eg, sleep and diet) on 18 items in 9 groups. SCL-90: Symptom Checklist 90.

**Figure 4:**
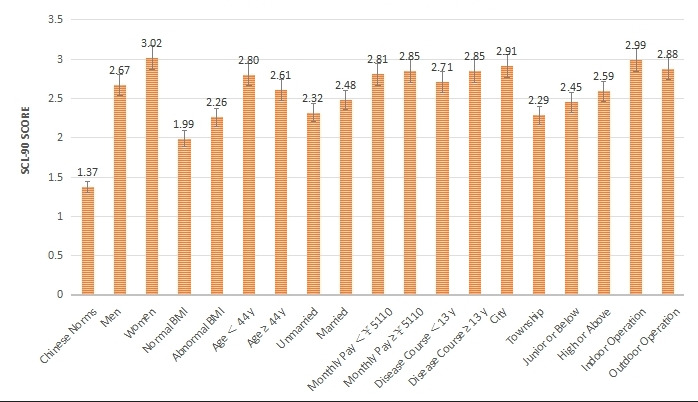
Comparison of 18 items in 9 groups and Chinese norms on total average score. SCL-90: Symptom Checklist 90.

**Figure 5:**
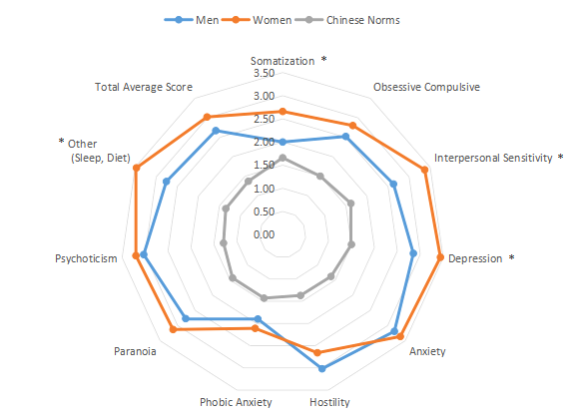
Intragroup comparison of gender. *P<.05.

The SCL-90 was used to group and score the mental state of 827 patients with AR (Table 1 and Figures 1-4). The intragroup comparison by gender (male: n=396; female: n=431) is shown in Table 1 and Figure 5.

**Figure 6:**
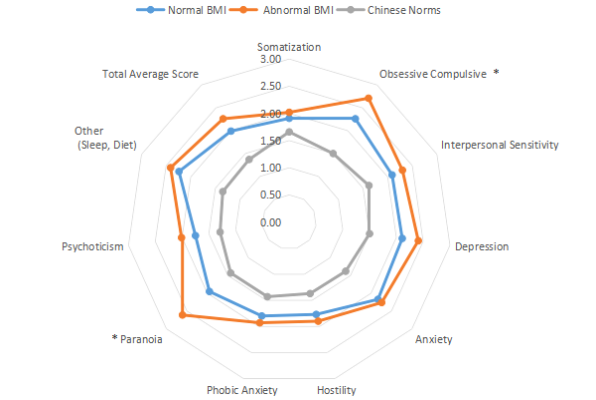
Intragroup comparison of BMI. *P<.05.

**Figure 7:**
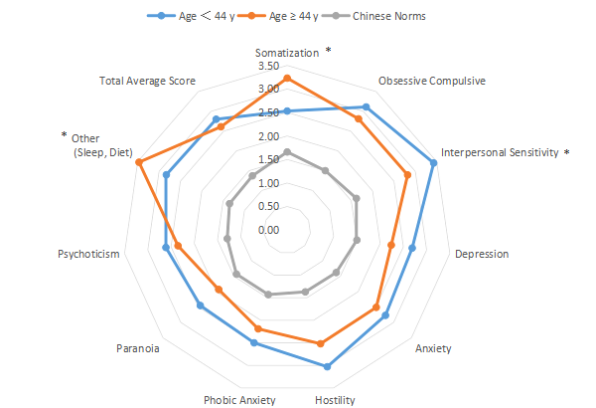
Intragroup comparison of age. *P<.05.

**Figure 8:**
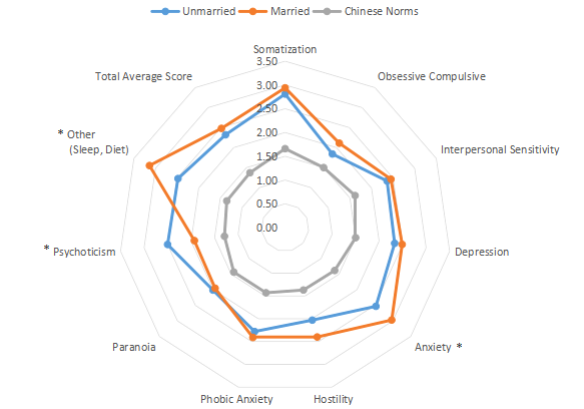
Intragroup comparison of marital status. *P<.05.

**Figure 9:**
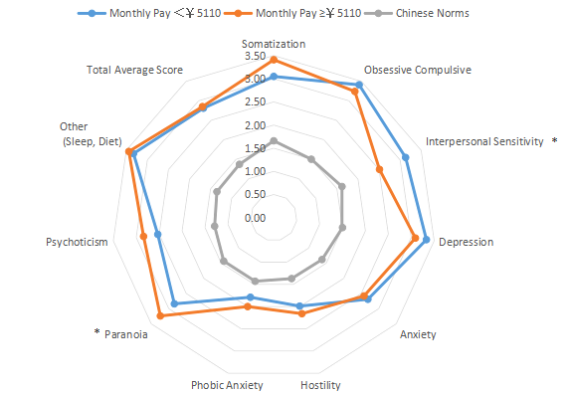
Intragroup comparison of monthly salary. *P<.05.

**Figure 10:**
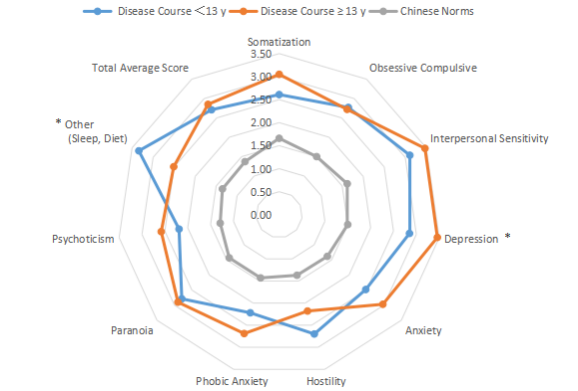
Intragroup comparison of disease duration. *P<.05.

The intragroup comparison of BMI is shown in Table 1 and Figure 6). In accordance with the WHO criteria for BMI [10], patients were divided into two groups: normal (18.5~24.9; n=531) and abnormal (<18.5 or ≥25; n=296). The intragroup comparison of age is shown in Table 1 and Figure 7. In accordance with the WHO age classification criteria [11], patients were divided into two groups: ages <44 years (n=438) and ≥45 years (n=389). The intragroup comparison by marital status (unmarried: n=448; married: n=379) is shown in Table 1 and Figure 8). The intragroup comparison by monthly salary is shown in Table 1 and Figure 9. Based on the average monthly salary (¥5110, US $700) of China’s Chengdu City in 2016 [12], patients were divided into two groups: <¥5110 (US $700; n=391) and ≥¥5110 (US $700; n=436). The intragroup comparison by disease duration is shown in Table 1 and Figure 10). Patients were divided into two age groups: <13 years (n=451) and ≥13 years (n=376).

**Figure 11:**
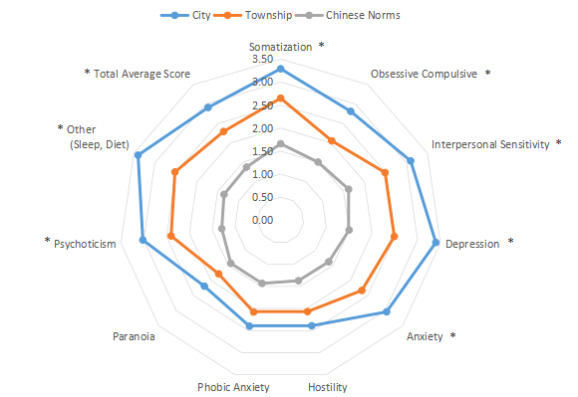
Intragroup comparison of living environment. *P<.05.

**Figure 12:**
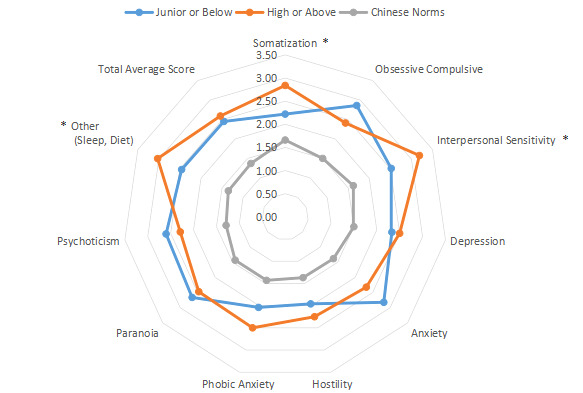
Intragroup comparison of education level. *P<.05.

**Figure 13:**
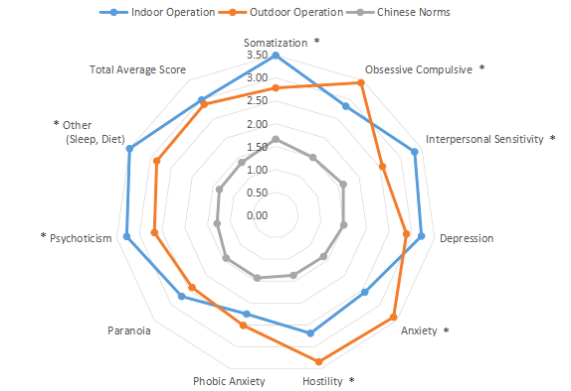
Intragroup comparison of working environment. *P<.05.

The intragroup comparison by living environment (urban or rural) is shown in Table 1 and Figure 11. The classification was made according to the Chinese administrative units [13], with those living in provinces, cities, and districts called “Urban” residents (n=471), and those living in townships, towns, and villages called “Rural” residents (n=356). The intragroup comparison by education level is shown in Table 1 and Figure 12. Patients were divided into two groups: “Junior high school and below” (n=379) and “Senior high school and above” (n=448). The intragroup comparison by working environment (indoors or outdoors) is shown in Table 1 and Figure 13). The Labor Law of the People’s Republic of China stipulates [14] that working hours should not exceed 8 hours a day. Based on this, the patients were divided into two groups: “Indoor” (average daily indoor working hours <4; n=475) and “Outdoor” (≥4 hours; n=352).

**Table 1:** The Symptom Checklist 90 was used to group and score the mental state of 827 patients with allergic rhinitis according to 9 classification criteria, namely, gender, BMI, age, marital status, monthly salary, disease duration, living environment (urban or rural), education level, and working environment (indoors or outdoors), and then all the scores were compared within groups.

Group		Somatization	Obsessive-compulsive	Interpersonal sensitivity	Depression	Anxiety	Hostility	Phobic anxiety	Paranoia	Psychoticism	Other (sleep, diet)	Total average score
**Gender**												
	Men, mean (SD)	2.00 (0.45)	2.52 (0.61)	2.63 (0.51)	2.85 (0.52)	3.19 (0.38)	3.02 (0.42)	1.90 (0.27)	2.77 (0.45)	3.03 (0.41)	2.76 (0.45)	2.67 (0.33)
	Women, mean (SD)	2.66 (0.51)	2.80 (0.57)	3.37 (0.47)	3.44 (0.57)	3.36 (0.67)	2.66 (0.35)	2.11 (0.60)	3.13 (0.48)	3.20 (0.32)	3.47 (0.36)	3.02 (0.45)
	*Z* score	–5.10	–1.22	–5.51	–4.86	–0.69	–1.70	–0.99	–1.75	–0.74	–5.28	–1.65^a^
	*P* value	*.03* ^b^	>.99	*.002*	*.04*	>.99	>.99	>.99	>.99	>.99	*.01*	>.99
**BMI**												
	Normal BMI, mean (SD)	1.91 (0.21)	2.26 (0.74)	2.09 (0.45)	2.11 (0.32)	2.17 (0.47)	1.77 (0.82)	1.80 (0.53)	1.95 (0.33)	1.75 (0.63)	2.24 (0.72)	1.99 (0.15)
	Abnormal BMI, mean (SD)	2.02 (0.33)	2.71 (0.63)	2.30 (0.41)	2.41 (0.88)	2.26 (0.53)	1.90 (0.30)	1.93 (0.96)	2.61 (0.71)	2.01 (0.48)	2.41 (0.40)	2.26 (0.38)
	*T* test (*df*)	0.61 (412)	4.10 (412)	2.26 (412)	3.24 (412)	0.36 (412)	1.38 (412)	1.25 (412)	5.29 (412)	2.95 (412)	1.77 (412)	3.13 (412)
	*P* value	.72	*.03*	.43	.20	.84	.64	.69	*.01*	.32	.51	.26
**Age (years)**												
	<44, mean (SD)	2.53 (0.33)	3.11 (0.42)	3.43 (0.38)	2.69 (0.28)	2.77 (0.50)	3.03 (0.35)	2.50 (0.44)	2.45 (0.67)	2.61 (0.66)	2.83 (0.52)	2.80 (0.41)
	≥44, mean (SD)	3.23 (0.52)	2.81 (0.45)	2.82 (0.53)	2.24 (0.43)	2.51 (0.41)	2.52 (0.47)	2.19 (0.51)	1.93 (0.23)	2.35 (0.52)	3.47 (0.38)	2.61 (0.33)
	*T* test (*df*)	5.22 (412)	1.70 (412)	5.06 (412)	3.36 (412)	1.08 (412)	4.09 (412)	1.70 (412)	4.22 (412)	1.10 (412)	5.10 (412)	–0.09 (412)
	*P* value	*.02*	>.99	*.03*	.16	>.99	.08	>.99	.05	>.99	*.03*	>.99
**Marital status**												
	Unmarried, mean (SD)	2.81 (0.26)	1.84 (0.56)	2.36 (0.44)	2.33 (0.70)	2.53 (0.80)	2.03 (0.66)	2.28 (0.34)	2.01 (0.36)	2.50 (0.31)	2.48 (0.12)	2.32 (3.70)
	Married, mean (SD)	2.94 (0.11)	2.11 (0.31)	2.45 (0.34)	2.49 (0.15)	2.97 (0.69)	2.40 (0.14)	2.41 (0.50)	1.95 (0.55)	1.93 (0.41)	3.14 (0.55)	2.48 (4.05)
	*Z* score	2.19	3.07	1.76	2.33	4.48	3.51	2.02	0.31	4.66	5.17	2.47
	*P* value	.48	.24	.77	.39	*.04*	.18	.59	.89	*.02*	*.001*	.41
**Monthly pay (** **¥)**												
	<5110^c^, mean (SD)	3.05 (0.22)	3.41 (0.65)	3.13 (0.27)	3.33 (0.45)	2.69 (0.44)	1.99 (0.82)	1.79 (0.82)	2.84 (0.55)	2.53 (0.90)	3.33 (0.51)	2.81 (0.94)
	≥5110, mean (SD)	3.41 (0.81)	3.24 (0.28)	2.51 (0.62)	3.09 (0.24)	2.58 (0.71)	2.16 (0.60)	2.00 (0.30)	3.24 (0.14)	2.84 (0.75)	3.44 (0.75)	2.85 (0.15)
	*Z* score	–3.88	–1.93	–4.93	–2.91	–1.48	–2.08	–2.65	–4.02	–3.46	–1.52	–1.77
	*P* value	.18	.55	*.02*	.33	.64	.49	.41	*.04*	.27	.60	>.99
**Disease course**												
	<13 years, mean (SD)	2.61 (0.76)	2.77 (0.50)	3.11 (0.33)	2.85 (0.38)	2.48 (0.27)	2.70 (0.22)	2.22 (0.50)	2.79 (0.36)	2.19 (0.29)	3.34 (0.55)	2.71 (0.60)
	≥13 years, mean (SD)	3.05 (0.41)	2.72 (0.38)	3.47 (0.70)	3.46 (0.59)	2.97 (0.41)	2.18 (0.34)	2.69 (0.39)	2.90 (0.88)	2.58 (0.58)	2.51 (0.24)	2.85 (0.39)
	*Z* score	1.38	0.22	0.90	4.98	1.49	2.37	1.42	0.57	0.96	5.44	0.64
	*P* value	.93	>.99	>.99	*.02*	.71	.65	.84	>.99	>.99	*.002*	>.99
**Living environment**												
	City, mean (SD)	3.29 (0.61)	2.81 (0.77)	3.10 (0.19)	3.40 (0.84)	3.04 (0.64)	2.39 (0.48)	2.40 (0.67)	2.19 (0.21)	3.02 (0.55)	3.40 (0.41)	2.91 (0.11)
	Township, mean (SD)	2.65 (0.50)	2.05 (0.38)	2.49 (0.67)	2.49 (0.70)	2.33 (0.51)	2.07 (0.46)	2.08 (0.40)	1.78 (0.18)	2.40 (0.77)	2.52 (0.77)	2.29 (0.40)
	*T* test (*df*)	4.88 (412)	5.25 (412)	4.57 (412)	5.91 (412)	5.09 (412)	1.83 (412)	1.79 (412)	2.55 (412)	4.63 (412)	5.55 (412)	–4.55 (412)
	*P* value	*.04*	*.01*	*.04*	*.003*	*.02*	.71	.79	.56	*.04*	*.001*	*.04*
**Educational level**												
	Junior or below, mean (SD)	2.22 (0.19)	2.86 (0.78)	2.52 (0.11)	2.33 (0.94)	2.82 (0.32)	1.96 (0.60)	2.04 (0.73)	2.66 (0.46)	2.60 (0.60)	2.46 (0.84)	2.45 (0.51)
	High or above, mean (SD)	2.84 (0.37)	2.41 (0.31)	3.19 (0.22)	2.50 (0.55)	2.32 (0.28)	2.25 (0.44)	2.50 (0.34)	2.47 (0.50)	2.29 (0.45)	3.03 (0.30)	2.59 (0.65)
	*Z* score	–4.82	–2.71	–4.90	–0.33	–3.38	–1.31	–2.94	–0.38	–1.46	–4.64	–0.27^a^
	*P* value	*.03*	.51	*.03*	>.99	.29	>.99	.46	>.99	.16	*.04*	>.99
**Working environment**												
	Indoors, mean (SD)	3.49 (0.64)	2.83 (0.35)	3.33 (0.30)	3.21 (0.29)	2.57 (0.93)	2.69 (0.56)	2.25 (0.25)	2.71 (0.10)	3.28 (0.60)	3.50 (0.85)	2.99 (0.48)
	Outdoors, mean (SD)	2.78 (0.46)	3.44 (0.40)	2.56 (0.68)	2.88 (0.14)	3.40 (0.50)	3.34 (0.88)	2.51 (0.15)	2.41 (0.32)	2.67 (0.31)	2.85 (0.37)	2.88 (0.72)
	*Z* score	5.31	4.96	5.79	1.51	6.22	5.08	1.02	1.11	4.89	5.00	0.04
	*P* value	*.01*	*.04*	*.002*	>.99	*.004*	*.03*	>.99	>.99	*.04*	*.03*	>.99

^a^These are *t* test values (*df* 412).

^b^Italics indicate statistical significance at the <.05 level.

^c^US $700.

### Intergroup Comparison

A comparison was made between the AR group and the Chinese norm, and the positive factors of the SCL-90 were extracted (Table 2). Among 827 patients, 124 (15%) had no mental health impairments, 176 (21.3%) had mild impairments, 474 (57.3%) had mild to moderate impairments, 41 (5%) had moderate to severe impairments, and 12 (1.4%) had severe impairments. The average score of the SCL-90 for all 827 patients was 2.64 (SD 0.25), which corresponded to the mild to moderate mental health impairments.

**Table 2:** Comparison between the AR group (study group) and the Chinese norm (healthy control group).

	AR^a^, mean (SD)	Chinese norm, mean (SD)	*Z* score	*P* value
Somatization	2.75 (0.43)	1.66 (0.28)	–3.15	>.99
Obsessive-compulsive	2.71 (0.22)	1.50 (0.89)	–3.87	.13
Interpersonal sensitivity	2.83 (0.37)	1.62 (0.61)	–3.99	.10
Depression	2.80 (0.51)	1.50 (0.59)	–5.51	*.01* ^b^
Anxiety	2.72 (0.40)	1.38 (0.43)	–6.03	*.002*
Hostility	2.39 (0.36)	1.37 (0.48)	–2.11	>.99
Phobic anxiety	2.20 (0.31)	1.43 (0.58)	–0.98	>.99
Paranoia	2.49 (0.62)	1.43 (0.57)	–2.74	>.99
Psychoticism	2.54 (0.56)	1.29 (0.42)	–4.61	*.02*
Other (sleep, diet)	2.95 (0.47)	1.35 (0.39)	–8.17	*<.001*
Total average score	2.64 (0.25)	1.37 (0.48)	–5.09	*.02*

^a^AR: allergic rhinitis.

^b^Italics indicate statistical significance at the <.05 level.

### Partial Correlation Analysis

Partial correlation analysis was performed on the 4 positive factors of the SCL-90 and the 4 symptoms of AR (Table 3). First, the VAS was used to evaluate the 4 AR symptoms separately. Nasal itching had 7 points, sneezing 8 points, clear discharge 8 points, and nasal congestion 6 points. Second, partial correlation analysis was made between the scores of the 4 symptoms and the 4 positive factors in Table 2 through the multiple regression statistical method by taking measurement data like BMI, age, monthly salary, and disease duration as the control variables.

**Table 3:** Partial correlation analysis between 4 positive factors of the SCL-90 and 4 symptoms of allergic rhinitis.

Positive factors of SCL-90^a^	Nasal itching		Sneezing		Clear discharge		Nasal congestion	
	*r*	*P* value	*r*	*P* value	*r*	*P* value	*r*	*P* value
Depression	0.13	.27	0.37	*.02* ^b^	0.47	*.001*	0.05	.70
Anxiety	0.42	*.01*	0.11	.33	0.20	.08	0.43	*.01*
Psychoticism	0.33	*.03*	0.40	*.01*	0.17	.14	0.18	.20
Other (sleep, diet)	0.15	.25	0.13	.26	0.30	*.04*	0.32	*.03*

^a^SCL-90: Symptom Checklist 90.

^b^Italics indicate statistical significance at the <.05 level.

## Discussion

### Principal Findings

Zheng et al [15] pointed out that 69.45% of patients with AR have depressive tendencies. While treating rhinitis, doctors should give “regular and habitual” comfort and follow-ups, and if necessary, should refer patients to psychologists. Muñoz-Cano et al [16] found by using the self-rating anxiety scale that 58.77% of AR patients’ anxiety could not be ignored, and their life happiness index decreased with a prolonged course of the disease. If patients did not receive active “packaged treatment” (understanding, care, counseling, therapy, follow-up) [17], they would turn from “unhealthily free” to “unfreely anxious” [18] and, in extreme cases, might even induce violence [19]. From 2011 to 2014, a total of 4 chief otolaryngologists were killed with a knife by patients in China. Therefore, it is imperative to strictly follow the “biological-psychological-social” international medical model and to have a comprehensive understanding of the association between AR and psychological disorders.

### Comparison to Prior Work

In the existing literature, despite many in-depth studies, there are few comprehensive studies in this regard due to the following reasons. First, part of the literature only made one-on-one retrospective analysis or targeted meta-analysis of AR with depression, anxiety, hostility, and other psychological disorders [20], while few used the SCL-90 scale for a comprehensive 10-factor study. Second, although some literature used the SCL-90 scale, they only investigated the patients within respective hospitals [21], resulting in small sample sizes that could only reflect the status of a district rather than a city. Third, some literature surveyed multiple hospitals with the SCL-90 scale, but the research teams were limited to the otolaryngology department only, where the cases were often collected by nurses and the doctors were responsible for the diagnosis, treatment, and final data analysis [22]. Such results would appear to be less professional and authoritative because of the lack of cooperation from psychologists and psychiatrists.

Therefore, this study addresses the above 3 shortcomings and makes the following 3 optimizations on the research design. First, the SCL-90 scale was introduced systematically, which has a good discriminating function as it includes 90 items such as consciousness, emotion, diet/sleep, sensation, and lifestyle, and comprehensively reflects the distribution characteristics of 10 psychological symptoms with 10 factors [23]. Second, this study specifically covers the 5 most renowned hospitals in Chengdu, China, which not only enlarges the sample size but also takes into account the geographical locations in all directions so that the data are more detailed and can more accurately reflect the real-world research of a city. Third, this study is fortunately sponsored by two National Natural Science Foundations, so we were able to invite psychologists and psychiatrists to carry out the experimental design and data analysis together, thus making the team configuration better and the results more authoritative.

### Limitations

To our knowledge, this is the first multicenter and interdisciplinary study of patients with AR. However, there are some limitations in this study. First, a few patients inevitably pretend to cooperate when filling out the questionnaire, which will affect the accuracy of the data. Second, psychological factors are greatly influenced by subjective factors, and the psychological state of patients at the time of filling in the form cannot reflect their later psychological state. Third, this study took place before the COVID-19 pandemic, but unfortunately, epidemic factors were not included in the study as a single factor. Since the outbreak of COVID-19 in Chengdu, China, the number of patients with mental diseases has increased greatly.

### Conclusions

The intergroup comparisons in this study were performed on 827 patients with AR in China’s Chengdu City versus the Chinese norm, which reveal mild to moderate psychological disorders. The scores for 4 factors, namely, depression, anxiety, psychosis, and other (sleep, diet), are statistically higher than the Chinese norm, indicating that the psychological disorders develop mainly into these 4 types among the patients with AR. Therefore, otolaryngologists should pay close attention to the above four psychological states of patients when assessing the curative efficacy of somatopathies. Specifically, this study comprises 9 intragroup comparisons and 2 intergroup comparisons. Among the 9 classification criteria for intragroup comparisons, the living environment (urban or rural) yielded 8 positive results; working environment (indoors or outdoors) yielded 7 positive results; gender yielded 4 positive results; age, marital status, and education level yielded 3 positive results each; and BMI, monthly salary, and disease duration yielded 2 positive results each. This demonstrates the influencing factors of AR patients’ physical and mental health and their proportions, as well as the importance of the “biological-psychological-social” medical model. Meanwhile, these results also remind the otolaryngologists in Chengdu to offer more care and follow-ups to the following target positive populations: women and those with abnormal BMI, aged ≥45 years, with a monthly salary <¥5110 (US $700), with a disease duration <13 years, residing in urban areas, with a high school or above education, and who are indoor laborers.

## Abbreviations

AR: allergic rhinitis
SCL-90: Symptom Checklist 90
VAS: visual analog scale
WHO: World Health Organization
